# Micro-climatic effects on plant phenolics at the community level in a Mediterranean savanna

**DOI:** 10.1038/s41598-020-71782-5

**Published:** 2020-09-08

**Authors:** Xoaquín Moreira, Luis Abdala-Roberts, M. Dolores Hidalgo-Galvez, Carla Vázquez-González, Ignacio M. Pérez-Ramos

**Affiliations:** 1 Misión Biológica de Galicia (MBG-CSIC), Apdo. 28, 36080 Pontevedra, Galicia, Spain; 2 Department of Tropical Ecology, Autonomous University of Yucatan, Apartado Postal 4-116, 97000 Itzimna, Mérida, Yucatan Mexico; 3 Institute of Natural Resources and Agrobiology of Seville (IRNAS-CSIC), 10 Reina Mercedes Avenue, 41012 Seville, Spain

**Keywords:** Ecology, Plant sciences

## Abstract

Research has shown that warming and drought change plant phenolics. However, much of this work has centered on the effects of individual abiotic stressors on single plant species rather than the concurrent effects of multiple stressors at the plant community level. To address this gap, we manipulated rainfall and air temperature to test for their individual and interactive effects on the expression of leaf phenolics at the community level for annual plant species occurring in two habitat types (under oak tree canopies or in open grasslands) in a Mediterranean savanna. We found that augmented temperature had a significant positive effect on the community-weighted mean of total phenolics whereas reduced rainfall had no effect. In addition, we found no evidence of interactive effects between climatic stressors and these patterns remained consistent across habitat types. Overall, this study points at increasing efforts to investigate the linkages between climate change and community-level shifts in plant secondary chemistry.

## Introduction

Secondary metabolites play a pivotal role in plant protection against biotic aggressors (e.g. herbivores, pathogens) and abiotic stresses (e.g. drought, warming, ultraviolet radiation), and also shape ecosystem processes via effects on decomposition and nutrient cycling which feedback to affect plant communities and food webs^1^. Notably, phenolic compounds such as simple phenols, phenolic acids (hydroxybenzoic acids and hydroxycinnamic acids), coumarins, polyphenols flavonoids (flavones, flavonols, isoflavonols, flavanones, flavanols, anthocyanins, betacyanins, betalains, betaxanthins, chalcones, etc.), and non-flavonoids (tannins, lignans, and stilbenes) are carbon-based secondary metabolites that are ubiquitous in plant tissues and broadly distributed throughout the plant kingdom^2^. Previous work has found that abiotic stressors such as increasing temperatures, drought, and salinity frequently increase the expression of secondary metabolites^3–7^, including phenolic compounds^8–14^. While much of this work has focused on the effects of individual abiotic stressors on plant phenolics, few studies have tested the effects of multiple stressors simultaneously^15^. This is a key aspect to consider since climatic factors are often coupled and exert interactive effects which make their joint consideration essential for understanding the effects of climate change on ecosystem function via changes in plant phenolics.

Most previous work has focused on the effects of abiotic forcing on the secondary metabolism of individual plant species^16^. While some of the species that have been studied are dominant and/or play important roles in ecosystem function, by focusing on single species rather than functional groups or entire communities it is not possible to infer beyond these taxa and gain an understanding of community-wide patterns. The few available studies that have tested for abiotic control over plant phenolics at the community level have done so by measuring responses by multiple co-occurring species and computing community-weighted trait means (i.e. trait values weighted by species abundances within individual spatial replicates^17^). This approach has the advantage over assessing effects individually for multiple species in that it summarizes variation across species which can then be more easily and directly linked to shifts in ecosystem function. Yet, more work is needed spanning different community and ecosystem types.

In this study, we individually manipulated rainfall (ambient vs. reduced rainfall) and air temperature (ambient vs. increased temperature) to test for their individual as well as interactive effects on the expression of leaf phenolics at the community level. We achieved this by computing community-weighted means for total phenolics (CWM_phenolics_) across a diverse group of annual plant species naturally growing in a Mediterranean savanna. In doing so, we seek to improve current understanding of how climate change stressors shape the secondary chemistry of plant communities.

## Results

**Figure 1:**
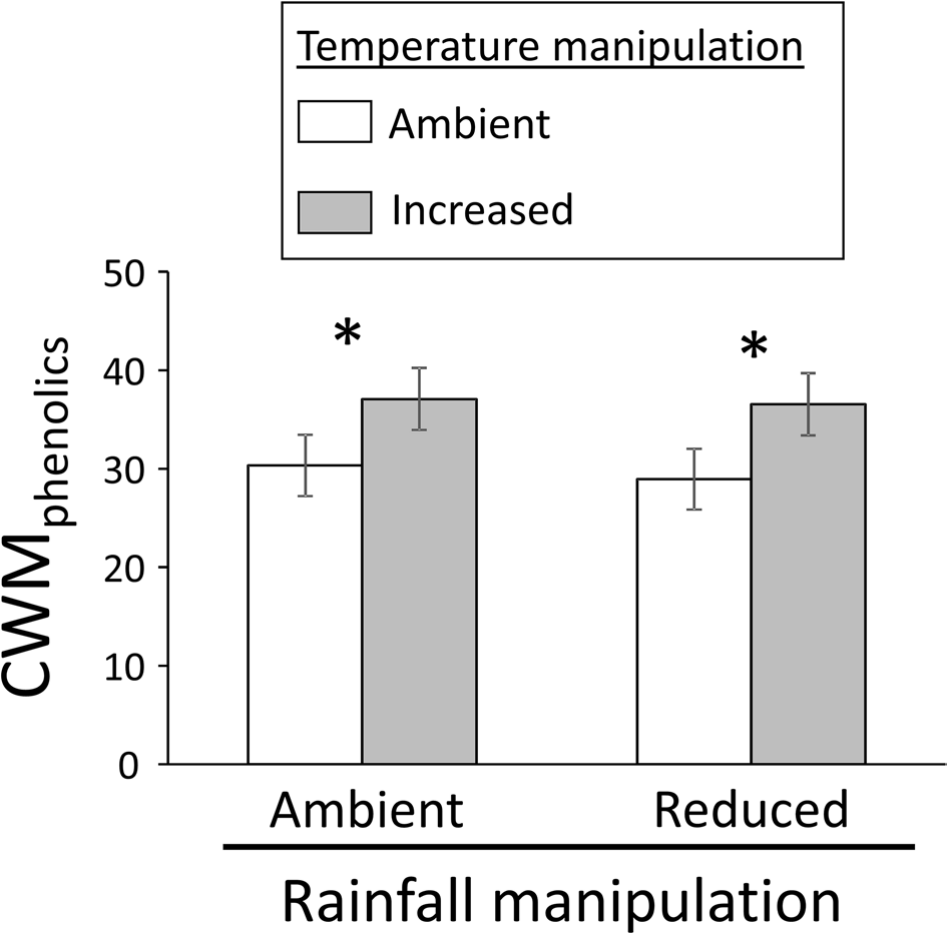
Effects of abiotic treatments on plant phenolics. Community-Weighted Means of leaf total phenolic concentration (CWM_phenolics_ in mg g^−1^ d.w.) in annual plant species growing under rainfall manipulations (two levels: ambient vs. reduced rainfall) and temperature manipulations (two levels: ambient vs. increased temperature). Bars are least square means ± s.e.m. (N = 31). Asterisks above the bars indicate significant (*P* < 0.05) differences between ambient and increased temperature within rainfall manipulation treatments.

We found no significant main effects of habitat type (open: 34.81 ± 2.54 mg g^−1^ d.w.; shaded: 31.64 ± 2.61 mg g^−1^ d.w.) or rainfall reduction (reduced: 32.75 ± 2.57 mg g^−1^ d.w.; ambient: 33.71 ± 2.58 mg g^−1^ d.w.) on CWM_phenolics_ (Table 1, Fig. 1). In contrast, the warming treatment exerted a significant effect on plant chemistry (Table 1, Fig. 1), with plots subjected to warming exhibiting a ca. 24% greater CWM_phenolics_ mean value (36.82 ± 2.59 mg g^−1^ d.w.) compared to those found under ambient temperature (29.63 ± 2.56 mg g^−1^ d.w.) (Table 1, Fig. 1). We found no evidence of significant two-way interactions between climatic treatments or between habitat type and the climatic treatments (Table 1). Likewise, the three-way habitat type × rainfall × temperature interaction was not significant either (Table 1).

**Table 1:** Results from general linear mixed model testing for the effects of habitat type (two levels: open grassland vs. under tree canopy), rainfall manipulation (two levels: ambient vs. reduced rainfall), temperature manipulation (two levels: ambient vs. increased temperature), and their two-way and three-way interactions (all main effects and interactions treated as fixed factors) on the community-weighted mean for total leaf phenolics.

	DF_num,den_	F-value	*P*
Habitat type (H)	1, 2	1.58	0.336
Rainfall (R)	1, 4	0.15	0.722
Temperature (T)	1, 111	8.12	**0.005**
H × R	1, 4	0.11	0.758
H × T	1, 111	0.84	0.361
R × T	1, 111	0.03	0.869
H × R × T	1, 111	0.12	0.731

We included site, the site × habitat type interaction and the site × habitat type × rainfall manipulation interaction as random factors in order to analyse the main factors habitat type and rainfall manipulation with the appropriate error terms. F-values, degrees of freedom (numerator, denominator), and associated significance levels (*P*) are shown. Significant effects (*P* < 0.05) are in bold.

The PERMANOVA test indicated a difference in plant species composition between years, but no effect of treatment or a significant treatment by year interaction (Table S2). This indicates that any such effects of abiotic manipulations on phenolic compounds did not occur via changes in plant composition.

## Discussion

Our results showed that warming significantly increased the community-level expression of phenolic compounds in annual herbs of the studied savanna ecosystem. This finding agrees with previous work on single species in arboreal and grassland ecosystems showing that air temperature increases of 1.5–4.0 °C boost the production of phenolic compounds in leaves or roots of grass species^18^, willows^19^, and birches^20^. The proposed mechanism is that phenolics act as antioxidants, either by enzymatic or direct radical scavenging mechanisms, which attenuate the harmful effects of active oxygen species produced within cells due to abiotic stresses^21^. Because we did not detect a treatment effect or temporal variation in its effect on species composition, it is likely that the warming effect was due to within-species phenotypic changes due to plasticity in response to this manipulation. On the other hand, rainfall manipulation did not affect CWM_phenolics_, which counters previous work showing increased production of phenolics under drought conditions^8–14^. One plausible explanation for this unexpected finding is that 2018 was a rainy year (approx. 600 mm) and therefore quadrats subjected to rainfall reduction probably experienced weak drought effects.

Our results also indicated no evidence of interactive (i.e. non-additive) effects between abiotic treatments, such that the observed positive effect of warming was of similar magnitude across rainfall levels. Despite such lack of interaction, this finding is important since few studies have examined the joint impact of multiple climatic stressors on plant phenolics and other secondary metabolites. In one of the few available studies, Orians et al.^15^ found that warming and drought had independent, additive effects on the expression of secondary metabolites (sorbitol, citrate and malate) in *Plantago lanceolata*. In our case, the weakened effect of drought due to higher-than average rainfall during the study could have also precluded interactive effects. At the same time, this finding could also indicate that warming effects remain consistent under increased rainfall.

While work focusing on effects on a single species has been highly valuable in describing the nature (sign) and variability (both intra- and inter-specific) of climatic effects on plant phenolics expression, efforts by individual studies to address these effects at the plant community-level are ultimately needed to increase inference. Our present work calls for research adopting a community-level perspective to climate change effects on plant secondary chemistry in order to establish more direct links between climate change, plant chemistry, and ecosystem processes. Considering these community-level changes in secondary chemistry at multiple spatial scales will shed light on scale-dependent processes or mechanisms driving observed patterns, whereas conducting longer-term measurements will allow to incorporate climate unpredictability, an important characteristic of future climate change.

## Methods

### Study area

This study was conducted at the Sierra Morena mountain range in Southwestern Spain (38° 22′ 50.64″ N, 4° 45′ 27.69″ W; Pozoblanco, Córdoba). This area has a continental-Mediterranean climate characterised by cold winters and hot, dry summers. Annual precipitation is 439 mm year^−1^ and mean annual temperature is 15.2 °C. The studied ecosystem is a “Mediterranean dehesa”, a savannah-type forest managed as a traditional sylvo-pastoral system, with an herbaceous stratum of native pasture and a tree layer of scattered oak trees (*Quercus ilex* L.). Tree density is 14.5 ± 1.3 trees ha^−1^, and the herbaceous layer is dominated by a diverse community of annual native species such as: *Hordeum murinum* (L.), *Senecio vulgaris* (L.), *Bromus madritensis* (L.), and *Sinapis alba* (L.), with a mean species richness of 9.6 ± 0.3 species m^−2^.

### Experimental design

**Figure 2:**
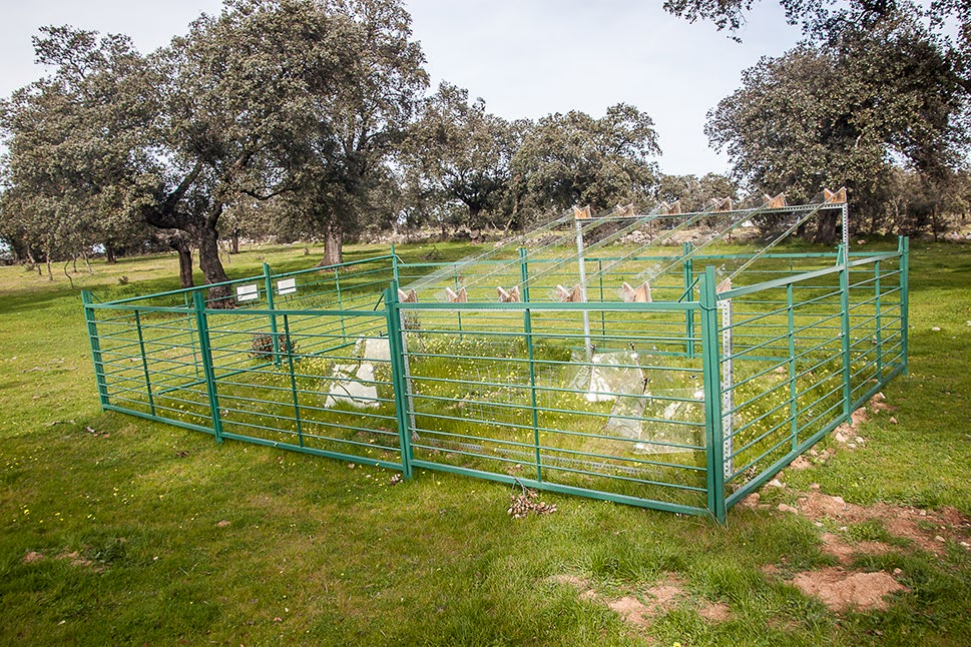
Experimental design. Picture showing open top chambers and rain-exclusion shelters used for our climate manipulations. Photo credit: Ignacio M. Pérez-Ramos.

In September 2016, we selected three sites separated by 4.3 ± 1.9 km with similar tree density, topography, slope, orientation, and soil type. At each site, we sampled two areas: a partially shaded site under *Q. ilex* canopy (under tree canopy) and a nearby open grassland site (open grassland). These two habitat types are characteristic of the dehesa ecosystem in the region. Then, within each area (i.e. habitat type) at each site we established six permanent 4 × 6 m (1.2 m high) fenced plots (N = 36 plots). Each plot was divided into two subplots (N = 72 subplots), one subplot was subjected to a rainfall reduction treatment whereas the other was exposed to ambient rainfall. Rainfall was reduced by placing a 2.5 × 2.5 m rain-exclusion shelter over one of the subplots (Fig. 2), resulting in a 30% reduction in annual rainfall^22^ as predicted by the IPCC for Mediterranean regions^23^. In addition, within each subplot we sampled two ca. 1-m^2^ quadrats (144 quadrats in total, i.e. 2 quadrats × 72 subplots): one was subjected to a warming treatment and the other was not manipulated (i.e. ambient temperature). Warming was achieved by placing a 40 × 50 × 32 cm (ca. 0.65 m^2^) hexagonal open top chamber of methacrylate material without UV filter (Faberplast, Madrid) at the centre of the quadrat (Fig. 2), increasing air temperature by ca. 2 °C compared to ambient temperature (17.5 ± 0.1 vs. 15.7 ± 0.1 °C, respectively). This matches the temperature increase for the study region by climatic forecasting models (SRES A-2 model by the Intergovernmental Panel on Climate Change^23^). Ambient air temperature was slightly lower (approx. 0.5 °C) in the shaded habitat than in the open habitat, but the experimental temperature increase was of similar magnitude in both cases. Temperature in each quadrat was measured with data loggers. Based on the above set-up, the experiment followed a randomized split–split plot design replicated across three sites, with habitat type as the whole factor (plots as replicates), rainfall manipulation as the split factor (subplots), and temperature manipulation as the split–split factor (quadrats).

### Sampling, measurements and chemical analyses

In April 2017 and April 2018, i.e. 1 and 2 years after establishment of climatic treatments, we identified all plant species and estimated their frequency within each quadrat. Then, in April 2018, after the second survey of species frequencies, we collected 3–4 fully expanded leaves from each plant for chemical analyses which were pooled to obtain a single sample per species and quadrat. Plant sampling was restricted to a central portion of 0.65 m^2^ in each quadrat (within chamber in the case of warming quadrats) as a function of the surface covered by and sampled within the hexagonal chamber placed in quadrats subjected to warming (see above). Due to lack of plant material, we only sampled 127 out of the 144 quadrats. In total, we collected 436 samples from 28 annual plant species (Table S1). Immediately after collection, we oven-dried leaves for 48 h at 40 °C, ground them with liquid nitrogen, and stored them at room temperature before conducting chemical analyses.

We determined total phenolic content colorimetrically by the Folin-Ciocalteu method^24,25^. Briefly, we extracted phenolics from 20 mg of plant tissue with 70% methanol in an ultrasonic bath for 15 min, followed by centrifugation. We determined total phenolic content colorimetrically by the Folin–Ciocalteu method in a Biorad 650 microplate reader (Bio-Rad Laboratories, PA, USA) at 740 nm, using tannic acid as standard. We performed three technical replicates of each sample in order to estimate variations due to the experimental procedure, and expressed concentrations based on dry weights (d.w.). We decided to use total phenolics as these could be measured across all species in order to address the goal of testing for community-level effects of climatic stressors on plant secondary chemistry.

### Statistical analyses

For each quadrat, we calculated the CWM_phenolics_ using the *weighted.mean* function in R software^26^. For this, we used phenolic values at the species level weighed by the abundance (i.e. frequency) of each species. We then performed linear mixed models using PROC MIXED in SAS 9.4 (SAS Institute, Cary, NC)^27^ to test for the effects of habitat type, rainfall manipulation, temperature manipulation, and their two-way and three-way interactions (all fixed factors) on CWM_phenolics_. We included site, the site × habitat type interaction, and the site × habitat type × rainfall manipulation interaction as random factors. The two- and three-way interaction interactions tested the main effect of habitat type and rainfall manipulation (respectively) with the appropriate error terms according to a split-split plot design. We log-transformed original values to achieve normality of residuals.

We also assessed whether climatic manipulations influenced the community-level expression of phenolic compounds through changes in plant composition with a PERMANOVA (‘vegan’ package in R) that included the effects of climatic treatment, year, and their interaction.

## Supplementary information

Supplementary Tables.
