# Supplementary material for: Micro-climatic effects on plant phenolics at the community level in a Mediterranean savanna
Source: Sci Rep. 2020 Sep 8;10:14757. doi: 10.1038/s41598-020-71782-5 (PMC7479144; doi:10.1038/s41598-020-71782-5)
Supplement: Supplementary file 1 — Supplementary Tables. [file 41598_2020_71782_MOESM1_ESM.docx]

**Micro-climatic effects on plant phenolics at the community level in a Mediterranean savanna**

Xoaquín Moreira^1*^, Luis Abdala-Roberts^2^, M. Dolores Hidalgo-Galvez^3^, Carla Vázquez-González^1^, and Ignacio M. Pérez-Ramos^3*^

^1^Misión Biológica de Galicia (MBG-CSIC), Apdo. 28, 36080 Pontevedra, Galicia, Spain

^2^Department of Tropical Ecology, Autonomous University of Yucatan, Apartado Postal 4-116, Itzimna. 97000. Merida, Yucatan, Mexico

^3^Institute of Natural Resources and Agrobiology of Seville (IRNAS-CSIC), 10 Reina Mercedes Avenue, 41012 Seville, Spain

^*^Corresponding author:

Email: [xmoreira1@gmail.com](mailto:xmoreira1@gmail.com) or [imperez@irnase.csic.es](mailto:imperez@irnase.csic.es)

Phone Number: +34 986854800 Fax Number: + 34 986841362

**Table S1.** Permutational analysis of variance analysis (PERMANOVA) testing for the effects of year (two levels: 2017, 2018), rainfall manipulation (two levels: ambient vs. reduced rainfall), temperature manipulation (two levels: ambient vs. increased temperature), and their two-way interactions on plant species composition. Analysis is based on Bray-Curtis distances of species frequency data, using 10,000 permutations. Pseudo F-values, degrees of freedom (numerator, denominator), and associated significance levels (*P*) are shown. Significant *P* values (*P* < 0.05) are in bold.

| **Source** | DF_num,den_ | Pseudo-F | *P* | |
| --- | --- | --- | --- | --- |
| Year (Y) | 1, 287 | 7.28 | | **<0.001** |
| Rainfall (R) | 1, 287 | 1.19 | | 0.291 |
| Temperature (T) | 1, 287 | 1.52 | | 0.146 |
| Y × R | 1, 287 | 0.64 | | 0.729 |
| Y × T | 1, 287 | 0.69 | | 0.686 |
| R × T | 1, 287 | 7.28 | | 0.944 |

**Table S2.** Data used for this paper. The experiment included three main treatments, namely: habitat type (two levels: open grassland vs. under tree canopy), rainfall manipulation (two levels: ambient vs. reduced rainfall) and temperature manipulation (two levels: ambient vs. increased temperature). These main treatments were replicated across three sites. Concentration of total phenolics were expressed in mg g^-1^ tissue on a dry weight basis. Relative frequency of each species (“Frequency”) was estimated as the number of individuals of this species / total number of individuals of all species.

| Site | Habitat | Rainfall | Temperature | Plant species | Phenolics | Frequency |
| --- | --- | --- | --- | --- | --- | --- |
| 3 | tree | ambient | ambient | *Malva parviflora* | 21.138 | 62.500 |
| 3 | tree | ambient | ambient | *Sinapis alba* | 29.337 | 37.500 |
| 3 | tree | reduced | ambient | *Capsella bursa pastoris* | 17.091 | 19.048 |
| 3 | tree | reduced | ambient | *Stellaria media* | 19.484 | 52.381 |
| 3 | tree | reduced | ambient | *Urtica urens* | 26.556 | 28.571 |
| 3 | tree | ambient | increased | *Sinapis alba* | 32.520 | 100.000 |
| 3 | tree | reduced | increased | *Stellaria media* | 20.827 | 60.000 |
| 3 | tree | reduced | increased | *Urtica urens* | 37.229 | 40.000 |
| 3 | grassland | ambient | ambient | *Senecio vulgaris* | 13.235 | 6.250 |
| 3 | grassland | ambient | ambient | *Sinapis alba* | 31.381 | 62.500 |
| 3 | grassland | reduced | ambient | *Calendula arvensis* | 26.070 | 9.091 |
| 3 | grassland | ambient | increased | *Sinapis alba* | 25.788 | 87.500 |
| 3 | tree | ambient | ambient | *Calendula arvensis* | 23.462 | 31.250 |
| 3 | tree | ambient | ambient | *Erodium moschatum* | 50.203 | 6.250 |
| 3 | tree | ambient | ambient | *Lamium amplexicaule* | 20.603 | 12.500 |
| 3 | tree | ambient | ambient | *Sonchus oleraceus* | 61.863 | 31.250 |
| 3 | tree | reduced | ambient | *Geranium dissectum* | 109.472 | 10.000 |
| 3 | tree | reduced | ambient | *Vicia lutea* | 16.854 | 90.000 |
| 3 | tree | ambient | increased | *Geranium dissectum* | 85.882 | 78.571 |
| 3 | tree | ambient | increased | *Sonchus oleraceus* | 28.662 | 21.429 |
| 3 | tree | reduced | increased | *Geranium dissectum* | 106.034 | 20.000 |
| 3 | tree | reduced | increased | *Senecio vulgaris* | 88.417 | 60.000 |
| 3 | grassland | ambient | ambient | *Hypochaeris glabra* | 42.032 | 5.405 |
| 3 | grassland | ambient | ambient | *Sinapis alba* | 22.533 | 13.514 |
| 3 | grassland | reduced | ambient | *Erodium moschatum* | 62.828 | 2.500 |
| 3 | grassland | reduced | ambient | *Hypochaeris glabra* | 31.412 | 17.500 |
| 3 | grassland | reduced | ambient | *Sinapis alba* | 23.087 | 75.000 |
| 3 | grassland | ambient | increased | *Sinapis alba* | 20.972 | 6.897 |
| 3 | grassland | reduced | increased | *Erodium moschatum* | 101.496 | 65.385 |
| 3 | grassland | reduced | increased | *Sinapis alba* | 19.184 | 7.692 |
| 3 | tree | ambient | ambient | *Calendula arvensis* | 36.434 | 9.091 |
| 3 | tree | ambient | ambient | *Erodium moschatum* | 63.153 | 2.273 |
| 3 | tree | ambient | ambient | *Ornithopus compressus* | 22.279 | 18.182 |
| 3 | tree | ambient | ambient | *Senecio vulgaris* | 25.278 | 4.545 |
| 3 | tree | ambient | ambient | *Sinapis alba* | 16.139 | 9.091 |
| 3 | tree | reduced | ambient | *Calendula arvensis* | 30.467 | 52.941 |
| 3 | tree | reduced | ambient | *Senecio vulgaris* | 19.102 | 17.647 |
| 3 | tree | reduced | ambient | *Sinapis alba* | 24.609 | 23.529 |
| 3 | tree | ambient | increased | *Calendula arvensis* | 28.443 | 41.667 |
| 3 | tree | ambient | increased | *Erodium moschatum* | 76.647 | 19.444 |
| 3 | tree | ambient | increased | *Lamium amplexicaule* | 36.535 | 8.333 |
| 3 | tree | ambient | increased | *Mibora minima* | 36.210 | 2.778 |
| 3 | tree | ambient | increased | *Ornithopus compressus* | 28.358 | 13.889 |
| 3 | tree | ambient | increased | *Senecio vulgaris* | 20.703 | 8.333 |
| 3 | tree | ambient | increased | *Sinapis alba* | 53.902 | 5.556 |
| 3 | tree | reduced | increased | *Calendula arvensis* | 12.680 | 16.129 |
| 3 | tree | reduced | increased | *Erodium moschatum* | 26.982 | 6.452 |
| 3 | tree | reduced | increased | *Lamium amplexicaule* | 31.494 | 22.581 |
| 3 | tree | reduced | increased | *Ornithopus compressus* | 22.580 | 3.226 |
| 3 | tree | reduced | increased | *Senecio vulgaris* | 17.419 | 45.161 |
| 3 | tree | reduced | increased | *Sinapis alba* | 23.820 | 6.452 |
| 3 | grassland | ambient | ambient | *Calendula arvensis* | 23.138 | 56.140 |
| 3 | grassland | ambient | ambient | *Erodium moschatum* | 74.324 | 1.754 |
| 3 | grassland | ambient | ambient | *Euphorbia exigua* | 50.690 | 1.754 |
| 3 | grassland | ambient | ambient | *Ornithopus compressus* | 29.576 | 1.754 |
| 3 | grassland | ambient | ambient | *Sinapis alba* | 27.208 | 36.842 |
| 3 | grassland | ambient | ambient | *Stachys arvensis* | 74.498 | 1.754 |
| 3 | grassland | reduced | ambient | *Calendula arvensis* | 40.700 | 14.286 |
| 3 | grassland | reduced | ambient | *Ornithopus compressus* | 40.573 | 4.082 |
| 3 | grassland | reduced | ambient | *Sinapis alba* | 35.918 | 73.469 |
| 3 | grassland | reduced | ambient | *Stachys arvensis* | 82.910 | 4.082 |
| 3 | grassland | ambient | increased | *Calendula arvensis* | 30.133 | 22.857 |
| 3 | grassland | ambient | increased | *Sinapis alba* | 24.412 | 62.857 |
| 3 | grassland | ambient | increased | *Stachys arvensis* | 71.608 | 8.571 |
| 3 | grassland | reduced | increased | *Calendula arvensis* | 63.487 | 11.905 |
| 3 | grassland | reduced | increased | *Cerastium glomeratum* | 19.110 | 4.762 |
| 3 | grassland | reduced | increased | *Euphorbia exigua* | 94.661 | 16.667 |
| 3 | grassland | reduced | increased | *Sinapis alba* | 36.113 | 57.143 |
| 3 | grassland | reduced | increased | *Stachys arvensis* | 77.065 | 2.381 |
| 3 | tree | ambient | ambient | *Sinapis alba* | 19.355 | 100.000 |
| 3 | tree | reduced | ambient | *Lamium amplexicaule* | 24.058 | 26.667 |
| 3 | tree | reduced | ambient | *Senecio vulgaris* | 9.687 | 60.000 |
| 3 | tree | reduced | ambient | *Stellaria media* | 16.078 | 13.333 |
| 3 | tree | ambient | increased | *Sinapis alba* | 17.609 | 100.000 |
| 3 | tree | reduced | increased | *Lamium amplexicaule* | 19.455 | 34.783 |
| 3 | tree | reduced | increased | *Senecio vulgaris* | 8.134 | 8.696 |
| 3 | tree | reduced | increased | *Stellaria media* | 67.125 | 56.522 |
| 3 | grassland | ambient | ambient | *Erodium moschatum* | 97.732 | 3.333 |
| 3 | grassland | ambient | ambient | *Hypochaeris glabra* | 51.284 | 3.333 |
| 3 | grassland | ambient | ambient | *Ornithopus compressus* | 35.424 | 6.667 |
| 3 | grassland | ambient | ambient | *Sinapis alba* | 22.448 | 53.333 |
| 3 | grassland | ambient | ambient | *Stachys arvensis* | 77.043 | 3.333 |
| 3 | grassland | reduced | ambient | *Hypochaeris glabra* | 33.605 | 12.000 |
| 3 | grassland | reduced | ambient | *Ornithopus compressus* | 31.373 | 28.000 |
| 3 | grassland | ambient | increased | *Ornithopus compressus* | 66.984 | 2.632 |
| 3 | grassland | ambient | increased | *Sinapis alba* | 53.450 | 50.000 |
| 3 | grassland | ambient | increased | *Stachys arvensis* | 80.378 | 44.737 |
| 3 | grassland | reduced | increased | *Mibora minima* | 23.734 | 38.462 |
| 3 | grassland | reduced | increased | *Ornithopus compressus* | 26.498 | 38.462 |
| 3 | grassland | reduced | increased | *Senecio vulgaris* | 52.953 | 7.692 |
| 3 | grassland | ambient | ambient | *Hypochaeris glabra* | 51.078 | 42.308 |
| 3 | grassland | ambient | ambient | *Ornithopus compressus* | 35.059 | 11.538 |
| 3 | grassland | reduced | ambient | *Erodium moschatum* | 92.321 | 15.385 |
| 3 | grassland | reduced | ambient | *Hypochaeris glabra* | 2.622 | 38.462 |
| 3 | grassland | reduced | ambient | *Ornithopus compressus* | 22.090 | 15.385 |
| 3 | grassland | reduced | ambient | *Senecio vulgaris* | 57.253 | 7.692 |
| 3 | grassland | reduced | ambient | *Spergula arvensis* | 28.065 | 7.692 |
| 3 | grassland | ambient | increased | *Hypochaeris glabra* | 61.341 | 20.000 |
| 3 | grassland | ambient | increased | *Ornithopus compressus* | 26.260 | 40.000 |
| 3 | grassland | ambient | increased | *Sinapis alba* | 29.567 | 40.000 |
| 3 | grassland | reduced | increased | *Ornithopus compressus* | 24.675 | 18.182 |
| 3 | grassland | reduced | increased | *Senecio vulgaris* | 53.985 | 18.182 |
| 3 | grassland | reduced | increased | *Sinapis alba* | 32.565 | 9.091 |
| 1 | tree | ambient | ambient | *Lamium amplexicaule* | 1.299 | 4.545 |
| 1 | tree | ambient | ambient | *Senecio vulgaris* | 2.920 | 81.818 |
| 1 | tree | ambient | ambient | *Senecio vulgaris* | 1.339 | 81.818 |
| 1 | tree | ambient | ambient | *Stellaria media* | 11.472 | 9.091 |
| 1 | tree | ambient | ambient | *Stellaria media* | 4.259 | 9.091 |
| 1 | tree | ambient | ambient | *Urtica urens* | 1.706 | 4.545 |
| 1 | tree | ambient | ambient | *Urtica urens* | 11.770 | 4.545 |
| 1 | tree | reduced | ambient | *Senecio vulgaris* | 2.394 | 57.895 |
| 1 | tree | reduced | ambient | *Sinapis alba* | 8.766 | 5.263 |
| 1 | tree | reduced | ambient | *Urtica urens* | 0.457 | 36.842 |
| 1 | tree | ambient | increased | *Erodium moschatum* | 51.630 | 12.500 |
| 1 | tree | ambient | increased | *Lamium amplexicaule* | 21.752 | 31.250 |
| 1 | tree | ambient | increased | *Senecio vulgaris* | 30.129 | 56.250 |
| 1 | tree | reduced | increased | *Geranium dissectum* | 85.025 | 45.833 |
| 1 | tree | reduced | increased | *Lamium amplexicaule* | 16.783 | 33.333 |
| 1 | tree | reduced | increased | *Urtica urens* | 3.736 | 20.833 |
| 1 | grassland | ambient | ambient | *Erodium moschatum* | 50.941 | 30.000 |
| 1 | grassland | ambient | ambient | *Erodium moschatum* | 16.698 | 30.000 |
| 1 | grassland | ambient | ambient | *Senecio vulgaris* | 2.648 | 10.000 |
| 1 | grassland | ambient | ambient | *Senecio vulgaris* | 2.862 | 10.000 |
| 1 | grassland | ambient | ambient | *Sinapis alba* | 25.297 | 60.000 |
| 1 | grassland | ambient | ambient | *Sinapis alba* | 14.645 | 60.000 |
| 1 | grassland | reduced | ambient | *Erodium moschatum* | 61.375 | 11.111 |
| 1 | grassland | reduced | ambient | *Senecio vulgaris* | 15.442 | 8.889 |
| 1 | grassland | reduced | ambient | *Sinapis alba* | 22.216 | 80.000 |
| 1 | grassland | ambient | increased | *Erodium moschatum* | 33.419 | 5.455 |
| 1 | grassland | ambient | increased | *Geranium dissectum* | 92.257 | 9.091 |
| 1 | grassland | ambient | increased | *Senecio vulgaris* | 9.057 | 23.636 |
| 1 | grassland | ambient | increased | *Sinapis alba* | 22.830 | 61.818 |
| 1 | grassland | reduced | increased | *Erodium moschatum* | 61.099 | 19.403 |
| 1 | grassland | reduced | increased | *Geranium dissectum* | 100.969 | 2.985 |
| 1 | grassland | reduced | increased | *Senecio vulgaris* | 20.478 | 34.328 |
| 1 | grassland | reduced | increased | *Sinapis alba* | 28.714 | 41.791 |
| 1 | grassland | reduced | increased | *Spergula arvensis* | 5.445 | 1.493 |
| 1 | tree | ambient | ambient | *Erodium moschatum* | 15.064 | 26.087 |
| 1 | tree | ambient | ambient | *Erodium moschatum* | 62.772 | 26.087 |
| 1 | tree | ambient | ambient | *Senecio vulgaris* | 37.099 | 56.522 |
| 1 | tree | ambient | ambient | *Sinapis alba* | 12.241 | 4.348 |
| 1 | tree | ambient | ambient | *Sinapis alba* | 23.456 | 4.348 |
| 1 | tree | ambient | ambient | *Urtica urens* | 2.837 | 6.522 |
| 1 | tree | reduced | ambient | *Erodium moschatum* | 47.156 | 23.684 |
| 1 | tree | reduced | ambient | *Sinapis alba* | 23.667 | 36.842 |
| 1 | tree | reduced | ambient | *Stellaria media* | 14.878 | 21.053 |
| 1 | tree | reduced | ambient | *Urtica urens* | 3.261 | 18.421 |
| 1 | tree | reduced | increased | *Capsella bursa pastoris* | 5.665 | 7.143 |
| 1 | tree | reduced | increased | *Erodium moschatum* | 52.587 | 92.857 |
| 1 | grassland | ambient | ambient | *Anthemis arvensis* | 64.319 | 21.154 |
| 1 | grassland | ambient | ambient | *Calendula arvensis* | 38.504 | 5.769 |
| 1 | grassland | ambient | ambient | *Erodium moschatum* | 53.512 | 3.846 |
| 1 | grassland | ambient | ambient | *Erodium moschatum* | 13.955 | 3.846 |
| 1 | grassland | ambient | ambient | *Sinapis alba* | 28.589 | 69.231 |
| 1 | grassland | ambient | ambient | *Sinapis alba* | 16.081 | 69.231 |
| 1 | grassland | reduced | ambient | *Anthemis arvensis* | 34.279 | 11.667 |
| 1 | grassland | reduced | ambient | *Erodium moschatum* | 61.956 | 15.000 |
| 1 | grassland | reduced | ambient | *Senecio vulgaris* | 24.416 | 13.333 |
| 1 | grassland | reduced | ambient | *Sinapis alba* | 26.176 | 60.000 |
| 1 | grassland | ambient | increased | *Calendula arvensis* | 30.898 | 14.286 |
| 1 | grassland | ambient | increased | *Erodium moschatum* | 49.700 | 42.857 |
| 1 | grassland | ambient | increased | *Sinapis alba* | 28.450 | 42.857 |
| 1 | grassland | reduced | increased | *Calendula arvensis* | 13.036 | 20.588 |
| 1 | grassland | reduced | increased | *Erodium moschatum* | 37.255 | 14.706 |
| 1 | grassland | reduced | increased | *Senecio vulgaris* | 11.421 | 2.941 |
| 1 | grassland | reduced | increased | *Sinapis alba* | 19.564 | 61.765 |
| 1 | tree | ambient | ambient | *Lamium amplexicaule* | 21.873 | 14.286 |
| 1 | tree | ambient | ambient | *Senecio vulgaris* | 3.836 | 71.429 |
| 1 | tree | ambient | ambient | *Senecio vulgaris* | 21.827 | 71.429 |
| 1 | tree | ambient | ambient | *Sinapis alba* | 14.359 | 14.286 |
| 1 | tree | ambient | ambient | *Sinapis alba* | 24.761 | 14.286 |
| 1 | tree | reduced | ambient | *Erodium moschatum* | 47.981 | 47.826 |
| 1 | tree | reduced | ambient | *Sinapis alba* | 23.578 | 50.000 |
| 1 | tree | ambient | increased | *Calendula arvensis* | 18.649 | 6.667 |
| 1 | tree | ambient | increased | *Erodium moschatum* | 50.716 | 60.000 |
| 1 | tree | ambient | increased | *Senecio vulgaris* | 24.034 | 33.333 |
| 1 | tree | reduced | increased | *Calendula arvensis* | 14.311 | 3.448 |
| 1 | tree | reduced | increased | *Erodium moschatum* | 41.576 | 17.241 |
| 1 | tree | reduced | increased | *Lamium amplexicaule* | 23.855 | 17.241 |
| 1 | tree | reduced | increased | *Sinapis alba* | 23.032 | 55.172 |
| 1 | grassland | ambient | ambient | *Cerastium glomeratum* | 3.093 | 44.444 |
| 1 | grassland | ambient | ambient | *Cerastium glomeratum* | 3.015 | 44.444 |
| 1 | grassland | ambient | ambient | *Ornithopus compressus* | 23.413 | 22.222 |
| 1 | grassland | ambient | ambient | *Spergula arvensis* | 6.874 | 33.333 |
| 1 | grassland | ambient | ambient | *Spergula arvensis* | 17.999 | 33.333 |
| 1 | grassland | reduced | ambient | *Senecio vulgaris* | 8.866 | 16.667 |
| 1 | grassland | reduced | ambient | *Sinapis alba* | 18.372 | 83.333 |
| 1 | grassland | ambient | increased | *Cerastium glomeratum* | 3.580 | 26.667 |
| 1 | grassland | ambient | increased | *Linaria spartea* | 39.184 | 6.667 |
| 1 | grassland | ambient | increased | *Ornithopus compressus* | 36.449 | 40.000 |
| 1 | grassland | reduced | increased | *Ornithopus compressus* | 24.664 | 100.000 |
| 1 | tree | ambient | ambient | *Capsella bursa pastoris* | 2.297 | 6.977 |
| 1 | tree | ambient | ambient | *Geranium dissectum* | 84.425 | 27.907 |
| 1 | tree | ambient | ambient | *Senecio vulgaris* | 16.542 | 37.209 |
| 1 | tree | ambient | ambient | *Sinapis alba* | 9.719 | 27.907 |
| 1 | tree | reduced | ambient | *Capsella bursa pastoris* | 1.817 | 14.286 |
| 1 | tree | reduced | ambient | *Poa annua* | 50.228 | 14.286 |
| 1 | tree | reduced | ambient | *Senecio vulgaris* | 12.180 | 23.810 |
| 1 | tree | reduced | ambient | *Stellaria media* | 19.338 | 14.286 |
| 1 | tree | ambient | increased | *Erodium moschatum* | 53.472 | 12.500 |
| 1 | tree | ambient | increased | *Geranium dissectum* | 89.849 | 12.500 |
| 1 | tree | ambient | increased | *Senecio vulgaris* | 23.929 | 65.625 |
| 1 | tree | reduced | increased | *Anthemis arvensis* | 24.862 | 6.061 |
| 1 | tree | reduced | increased | *Capsella bursa pastoris* | 3.442 | 18.182 |
| 1 | tree | reduced | increased | *Geranium dissectum* | 95.925 | 21.212 |
| 1 | tree | reduced | increased | *Senecio vulgaris* | 21.092 | 39.394 |
| 1 | grassland | ambient | ambient | *Anthemis arvensis* | 21.368 | 1.923 |
| 1 | grassland | ambient | ambient | *Erodium moschatum* | 25.842 | 1.923 |
| 1 | grassland | ambient | ambient | *Senecio vulgaris* | 15.444 | 15.385 |
| 1 | grassland | ambient | ambient | *Sinapis alba* | 22.010 | 69.231 |
| 1 | grassland | ambient | ambient | *Sinapis alba* | 24.390 | 69.231 |
| 1 | grassland | ambient | ambient | *Spergula arvensis* | 15.533 | 5.769 |
| 1 | grassland | ambient | ambient | *Spergula arvensis* | 1.914 | 5.769 |
| 1 | grassland | reduced | ambient | *Erodium moschatum* | 42.105 | 40.323 |
| 1 | grassland | reduced | ambient | *Senecio vulgaris* | 16.879 | 1.613 |
| 1 | grassland | reduced | ambient | *Sinapis alba* | 24.382 | 58.065 |
| 1 | grassland | ambient | increased | *Erodium moschatum* | 66.873 | 33.871 |
| 1 | grassland | ambient | increased | *Sinapis alba* | 40.600 | 58.065 |
| 1 | grassland | ambient | increased | *Spergula arvensis* | 5.346 | 6.452 |
| 1 | grassland | reduced | increased | *Calendula arvensis* | 32.508 | 1.724 |
| 1 | grassland | reduced | increased | *Erodium moschatum* | 39.700 | 36.207 |
| 1 | grassland | reduced | increased | *Sinapis alba* | 19.631 | 62.069 |
| 1 | tree | ambient | ambient | *Geranium dissectum* | 28.356 | 25.000 |
| 1 | tree | ambient | ambient | *Lamium amplexicaule* | 13.189 | 8.333 |
| 1 | tree | ambient | ambient | *Senecio vulgaris* | 14.223 | 66.667 |
| 1 | tree | reduced | ambient | *Senecio vulgaris* | 7.935 | 100.000 |
| 1 | tree | ambient | increased | *Calendula arvensis* | 24.860 | 23.077 |
| 1 | tree | ambient | increased | *Senecio vulgaris* | 11.680 | 7.692 |
| 1 | tree | reduced | increased | *Anthemis cotula* | 15.549 | 66.667 |
| 1 | tree | reduced | increased | *Erodium moschatum* | 44.252 | 33.333 |
| 1 | tree | ambient | ambient | *Anthemis arvensis* | 79.083 | 39.063 |
| 1 | grassland | ambient | ambient | *Calendula arvensis* | 41.652 | 3.125 |
| 1 | grassland | ambient | ambient | *Erodium moschatum* | 61.307 | 3.125 |
| 1 | grassland | ambient | ambient | *Euphorbia exigua* | 82.736 | 10.938 |
| 1 | grassland | ambient | ambient | *Ornithopus compressus* | 19.468 | 4.688 |
| 1 | grassland | ambient | ambient | *Sinapis alba* | 23.418 | 39.063 |
| 1 | grassland | reduced | ambient | *Anthemis arvensis* | 47.055 | 23.404 |
| 1 | grassland | reduced | ambient | *Euphorbia exigua* | 107.781 | 2.128 |
| 1 | grassland | reduced | ambient | *Sinapis alba* | 32.307 | 57.447 |
| 1 | grassland | ambient | increased | *Anthemis arvensis* | 94.891 | 16.901 |
| 1 | grassland | ambient | increased | *Ornithopus compressus* | 34.051 | 2.817 |
| 1 | grassland | ambient | increased | *Sinapis alba* | 30.195 | 43.662 |
| 1 | grassland | reduced | increased | *Anthemis arvensis* | 23.364 | 15.152 |
| 1 | grassland | reduced | increased | *Ornithopus compressus* | 28.567 | 13.636 |
| 1 | grassland | reduced | increased | *Senecio vulgaris* | 65.158 | 10.606 |
| 1 | grassland | reduced | increased | *Sinapis alba* | 26.554 | 40.909 |
| 1 | tree | ambient | ambient | *Medicago polymorpha* | 24.784 | 100.000 |
| 1 | tree | reduced | ambient | *Erodium moschatum* | 48.991 | 11.111 |
| 1 | tree | reduced | ambient | *Medicago polymorpha* | 30.135 | 77.778 |
| 1 | grassland | ambient | ambient | *Anthemis arvensis* | 4.575 | 43.182 |
| 1 | grassland | ambient | ambient | *Anthemis arvensis* | 30.257 | 43.182 |
| 1 | grassland | ambient | ambient | *Calendula arvensis* | 29.903 | 2.273 |
| 1 | grassland | ambient | ambient | *Euphorbia exigua* | 41.806 | 4.545 |
| 1 | grassland | ambient | ambient | *Sinapis alba* | 25.990 | 50.000 |
| 1 | grassland | reduced | ambient | *Anthemis arvensis* | 30.717 | 30.189 |
| 1 | grassland | reduced | ambient | *Erodium moschatum* | 37.315 | 1.887 |
| 1 | grassland | reduced | ambient | *Euphorbia exigua* | 78.104 | 7.547 |
| 1 | grassland | reduced | ambient | *Ornithopus compressus* | 22.516 | 3.774 |
| 1 | grassland | reduced | ambient | *Sinapis alba* | 21.163 | 47.170 |
| 1 | grassland | ambient | increased | *Anagallis arvensis* | 40.666 | 1.449 |
| 1 | grassland | ambient | increased | *Anthemis arvensis* | 28.417 | 28.986 |
| 1 | grassland | ambient | increased | *Euphorbia exigua* | 69.933 | 8.696 |
| 1 | grassland | ambient | increased | *Sinapis alba* | 23.363 | 50.725 |
| 1 | grassland | reduced | increased | *Anthemis arvensis* | 9.787 | 13.462 |
| 1 | grassland | reduced | increased | *Erodium moschatum* | 88.194 | 11.538 |
| 2 | tree | ambient | ambient | *Erodium moschatum* | 62.042 | 30.000 |
| 2 | tree | ambient | ambient | *Geranium dissectum* | 81.938 | 30.000 |
| 2 | tree | ambient | ambient | *Senecio vulgaris* | 12.977 | 20.000 |
| 2 | tree | ambient | ambient | *Urtica urens* | 38.017 | 20.000 |
| 2 | tree | reduced | ambient | *Geranium dissectum* | 63.270 | 58.824 |
| 2 | tree | reduced | ambient | *Senecio vulgaris* | 9.137 | 3.922 |
| 2 | tree | reduced | ambient | *Stellaria media* | 11.702 | 37.255 |
| 2 | tree | ambient | increased | *Erodium moschatum* | 55.979 | 25.806 |
| 2 | tree | ambient | increased | *Geranium dissectum* | 76.352 | 35.484 |
| 2 | tree | ambient | increased | *Lamarckia aurea* | 30.152 | 6.452 |
| 2 | tree | ambient | increased | *Senecio vulgaris* | 9.325 | 19.355 |
| 2 | tree | reduced | increased | *Erodium moschatum* | 25.596 | 30.769 |
| 2 | tree | reduced | increased | *Geranium dissectum* | 43.609 | 26.923 |
| 2 | tree | reduced | increased | *Senecio vulgaris* | 7.749 | 38.462 |
| 2 | grassland | ambient | ambient | *Cerastium glomeratum* | 0.635 | 40.000 |
| 2 | grassland | ambient | ambient | *Geranium dissectum* | 109.859 | 45.000 |
| 2 | grassland | ambient | ambient | *Sinapis alba* | 23.339 | 15.000 |
| 2 | grassland | reduced | ambient | *Cerastium glomeratum* | 1.507 | 40.816 |
| 2 | grassland | reduced | ambient | *Erodium moschatum* | 77.996 | 14.286 |
| 2 | grassland | reduced | ambient | *Geranium dissectum* | 113.188 | 12.245 |
| 2 | grassland | reduced | ambient | *Sinapis alba* | 19.185 | 10.204 |
| 2 | grassland | ambient | increased | *Erodium moschatum* | 61.698 | 3.704 |
| 2 | grassland | ambient | increased | *Geranium dissectum* | 96.421 | 29.630 |
| 2 | grassland | ambient | increased | *Sinapis alba* | 16.302 | 66.667 |
| 2 | grassland | reduced | increased | *Cerastium glomeratum* | 6.903 | 10.000 |
| 2 | grassland | reduced | increased | *Erodium moschatum* | 65.952 | 13.333 |
| 2 | grassland | reduced | increased | *Sinapis alba* | 22.219 | 50.000 |
| 2 | tree | ambient | ambient | *Erodium moschatum* | 44.403 | 68.000 |
| 2 | tree | ambient | ambient | *Geranium dissectum* | 56.170 | 4.000 |
| 2 | tree | ambient | ambient | *Senecio vulgaris* | 8.879 | 8.000 |
| 2 | tree | ambient | ambient | *Sinapis alba* | 42.672 | 16.000 |
| 2 | tree | reduced | ambient | *Capsella bursa pastoris* | 16.090 | 3.571 |
| 2 | tree | reduced | ambient | *Erodium moschatum* | 30.031 | 28.571 |
| 2 | tree | reduced | ambient | *Geranium dissectum* | 27.082 | 7.143 |
| 2 | tree | reduced | ambient | *Lamium amplexicaule* | 31.208 | 7.143 |
| 2 | tree | reduced | ambient | *Senecio vulgaris* | 7.505 | 17.857 |
| 2 | tree | reduced | ambient | *Stellaria media* | 13.435 | 21.429 |
| 2 | tree | ambient | increased | *Erodium moschatum* | 60.675 | 42.105 |
| 2 | tree | ambient | increased | *Geranium dissectum* | 85.070 | 47.368 |
| 2 | tree | ambient | increased | *Stellaria media* | 15.170 | 10.526 |
| 2 | tree | reduced | increased | *Capsella bursa pastoris* | 21.339 | 2.778 |
| 2 | tree | reduced | increased | *Erodium moschatum* | 37.974 | 25.000 |
| 2 | tree | reduced | increased | *Geranium dissectum* | 32.806 | 13.889 |
| 2 | tree | reduced | increased | *Lamium amplexicaule* | 27.530 | 16.667 |
| 2 | tree | reduced | increased | *Senecio vulgaris* | 6.347 | 30.556 |
| 2 | tree | reduced | increased | *Stellaria media* | 20.763 | 11.111 |
| 2 | grassland | ambient | ambient | *Cerastium glomeratum* | 12.257 | 35.000 |
| 2 | grassland | ambient | ambient | *Erodium moschatum* | 37.471 | 10.000 |
| 2 | grassland | ambient | ambient | *Geranium dissectum* | 83.399 | 45.000 |
| 2 | grassland | ambient | ambient | *Senecio vulgaris* | 28.816 | 7.500 |
| 2 | grassland | ambient | ambient | *Sinapis alba* | 21.297 | 2.500 |
| 2 | grassland | reduced | ambient | *Cerastium glomeratum* | 15.387 | 12.821 |
| 2 | grassland | reduced | ambient | *Erodium moschatum* | 37.068 | 51.282 |
| 2 | grassland | reduced | ambient | *Geranium dissectum* | 48.969 | 33.333 |
| 2 | grassland | reduced | ambient | *Senecio vulgaris* | 21.640 | 2.564 |
| 2 | grassland | ambient | increased | *Cerastium glomeratum* | 13.355 | 3.571 |
| 2 | grassland | ambient | increased | *Erodium moschatum* | 43.098 | 60.714 |
| 2 | grassland | ambient | increased | *Geranium dissectum* | 45.685 | 10.714 |
| 2 | grassland | ambient | increased | *Sinapis alba* | 16.793 | 21.429 |
| 2 | grassland | reduced | increased | *Erodium moschatum* | 29.687 | 6.250 |
| 2 | grassland | reduced | increased | *Senecio vulgaris* | 28.050 | 12.500 |
| 2 | tree | ambient | ambient | *Geranium dissectum* | 38.227 | 15.000 |
| 2 | tree | ambient | ambient | *Stellaria media* | 12.045 | 25.000 |
| 2 | tree | ambient | ambient | *Urtica urens* | 20.768 | 60.000 |
| 2 | tree | reduced | ambient | *Erodium moschatum* | 30.633 | 5.000 |
| 2 | tree | reduced | ambient | *Geranium dissectum* | 59.912 | 87.500 |
| 2 | tree | reduced | ambient | *Urtica urens* | 1.639 | 7.500 |
| 2 | tree | ambient | increased | *Geranium dissectum* | 36.995 | 21.053 |
| 2 | tree | ambient | increased | *Lamium amplexicaule* | 34.264 | 21.053 |
| 2 | tree | ambient | increased | *Urtica urens* | 8.126 | 57.895 |
| 2 | tree | reduced | increased | *Calendula arvensis* | 10.059 | 28.571 |
| 2 | tree | reduced | increased | *Erodium moschatum* | 37.498 | 9.524 |
| 2 | tree | reduced | increased | *Geranium dissectum* | 50.985 | 47.619 |
| 2 | tree | reduced | increased | *Senecio vulgaris* | 8.153 | 9.524 |
| 2 | tree | reduced | increased | *Urtica urens* | 5.836 | 4.762 |
| 2 | grassland | ambient | ambient | *Calendula arvensis* | 12.756 | 5.455 |
| 2 | grassland | ambient | ambient | *Cerastium glomeratum* | 17.927 | 9.091 |
| 2 | grassland | ambient | ambient | *Geranium dissectum* | 81.799 | 40.000 |
| 2 | grassland | ambient | ambient | *Senecio vulgaris* | 13.521 | 1.818 |
| 2 | grassland | ambient | ambient | *Sinapis alba* | 18.616 | 40.000 |
| 2 | grassland | reduced | ambient | *Calendula arvensis* | 35.582 | 58.065 |
| 2 | grassland | reduced | ambient | *Cerastium glomeratum* | 6.741 | 25.806 |
| 2 | grassland | reduced | ambient | *Geranium dissectum* | 101.423 | 16.129 |
| 2 | grassland | ambient | increased | *Calendula arvensis* | 15.664 | 2.703 |
| 2 | grassland | ambient | increased | *Cerastium glomeratum* | 10.797 | 43.243 |
| 2 | grassland | ambient | increased | *Geranium dissectum* | 71.375 | 37.838 |
| 2 | grassland | ambient | increased | *Sinapis alba* | 17.229 | 5.405 |
| 2 | grassland | reduced | increased | *Erodium moschatum* | 47.852 | 89.474 |
| 2 | grassland | reduced | increased | *Geranium dissectum* | 95.530 | 5.263 |
| 2 | tree | ambient | ambient | *Calendula arvensis* | 22.230 | 37.037 |
| 2 | tree | ambient | ambient | *Erodium moschatum* | 52.094 | 11.111 |
| 2 | tree | ambient | ambient | *Geranium dissectum* | 71.992 | 33.333 |
| 2 | tree | ambient | ambient | *Senecio vulgaris* | 13.082 | 18.519 |
| 2 | tree | reduced | ambient | *Erodium moschatum* | 42.587 | 60.714 |
| 2 | tree | reduced | ambient | *Geranium dissectum* | 65.134 | 21.429 |
| 2 | tree | reduced | ambient | *Lamium amplexicaule* | 27.008 | 17.857 |
| 2 | tree | ambient | increased | *Erodium moschatum* | 59.204 | 26.471 |
| 2 | tree | ambient | increased | *Lamium amplexicaule* | 41.755 | 2.941 |
| 2 | tree | ambient | increased | *Senecio vulgaris* | 30.580 | 8.824 |
| 2 | tree | reduced | increased | *Calendula arvensis* | 13.209 | 9.091 |
| 2 | tree | reduced | increased | *Erodium moschatum* | 28.509 | 54.545 |
| 2 | tree | reduced | increased | *Geranium dissectum* | 56.929 | 15.152 |
| 2 | grassland | ambient | ambient | *Calendula arvensis* | 25.776 | 21.429 |
| 2 | grassland | ambient | ambient | *Cerastium glomeratum* | 1.518 | 4.762 |
| 2 | grassland | ambient | ambient | *Geranium dissectum* | 94.986 | 14.286 |
| 2 | grassland | ambient | ambient | *Senecio vulgaris* | 47.388 | 7.143 |
| 2 | grassland | ambient | ambient | *Sinapis alba* | 15.730 | 42.857 |
| 2 | grassland | reduced | ambient | *Erodium moschatum* | 49.491 | 40.000 |
| 2 | grassland | reduced | ambient | *Geranium dissectum* | 64.203 | 5.714 |
| 2 | grassland | reduced | ambient | *Senecio vulgaris* | 26.128 | 7.143 |
| 2 | grassland | reduced | ambient | *Sinapis alba* | 20.879 | 20.000 |
| 2 | grassland | ambient | increased | *Calendula arvensis* | 35.721 | 25.714 |
| 2 | grassland | ambient | increased | *Cerastium glomeratum* | 6.707 | 2.857 |
| 2 | grassland | ambient | increased | *Geranium dissectum* | 113.697 | 14.286 |
| 2 | grassland | reduced | increased | *Cerastium glomeratum* | 9.544 | 17.308 |
| 2 | grassland | reduced | increased | *Erodium moschatum* | 51.756 | 51.923 |
| 2 | grassland | reduced | increased | *Senecio vulgaris* | 47.545 | 17.308 |
| 2 | grassland | reduced | increased | *Sinapis alba* | 23.376 | 13.462 |
| 2 | tree | reduced | ambient | *Erodium moschatum* | 36.249 | 22.414 |
| 2 | tree | reduced | ambient | *Geranium dissectum* | 80.423 | 15.517 |
| 2 | tree | reduced | ambient | *Lamium amplexicaule* | 32.979 | 12.069 |
| 2 | tree | reduced | ambient | *Senecio vulgaris* | 19.075 | 5.172 |
| 2 | tree | reduced | ambient | *Stellaria media* | 17.912 | 27.586 |
| 2 | tree | ambient | increased | *Calendula arvensis* | 19.776 | 66.667 |
| 2 | tree | ambient | increased | *Erodium moschatum* | 55.052 | 16.667 |
| 2 | tree | ambient | increased | *Geranium dissectum* | 51.192 | 7.407 |
| 2 | tree | ambient | increased | *Lamium amplexicaule* | 47.611 | 1.852 |
| 2 | tree | ambient | increased | *Senecio vulgaris* | 14.429 | 7.407 |
| 2 | tree | reduced | increased | *Erodium moschatum* | 55.418 | 33.333 |
| 2 | tree | reduced | increased | *Lamium amplexicaule* | 36.786 | 11.111 |
| 2 | tree | reduced | increased | *Senecio vulgaris* | 15.598 | 33.333 |
| 2 | grassland | ambient | ambient | *Cerastium glomeratum* | 2.696 | 7.692 |
| 2 | grassland | ambient | ambient | *Geranium dissectum* | 61.825 | 48.718 |
| 2 | grassland | ambient | ambient | *Parentucelia latifolia* | 8.080 | 28.205 |
| 2 | grassland | ambient | ambient | *Senecio vulgaris* | 15.015 | 10.256 |
| 2 | grassland | reduced | ambient | *Cerastium glomeratum* | 6.769 | 32.653 |
| 2 | grassland | reduced | ambient | *Geranium dissectum* | 74.998 | 20.408 |
| 2 | grassland | reduced | ambient | *Parentucelia latifolia* | 29.432 | 12.245 |
| 2 | grassland | reduced | ambient | *Poa bulbosa* | 38.783 | 8.163 |
| 2 | grassland | ambient | increased | *Geranium dissectum* | 84.679 | 21.739 |
| 2 | grassland | ambient | increased | *Senecio vulgaris* | 9.593 | 43.478 |
| 2 | grassland | reduced | increased | *Geranium dissectum* | 50.764 | 11.111 |
| 2 | grassland | reduced | increased | *Senecio vulgaris* | 15.376 | 11.111 |
| 2 | tree | ambient | ambient | *Calendula arvensis* | 17.888 | 39.535 |
| 2 | 35 | ambient | ambient | *Geranium dissectum* | 46.786 | 4.651 |
| 2 | 35 | ambient | ambient | *Lamium amplexicaule* | 24.432 | 6.977 |
| 2 | 35 | ambient | ambient | *Senecio vulgaris* | 7.837 | 16.279 |
| 2 | 35 | ambient | ambient | *Stellaria media* | 10.600 | 11.628 |
| 2 | 35 | ambient | ambient | *Urtica urens* | 7.420 | 18.605 |
| 2 | 35 | reduced | ambient | *Urtica urens* | 8.899 | 100.000 |
| 2 | 35 | ambient | increased | *Geranium dissectum* | 26.750 | 44.444 |
| 2 | 35 | ambient | increased | *Lamium amplexicaule* | 16.222 | 22.222 |
| 2 | 35 | ambient | increased | *Urtica urens* | 7.399 | 33.333 |
| 2 | 35 | reduced | increased | *Geranium dissectum* | 18.663 | 50.000 |
| 2 | 35 | reduced | increased | *Urtica urens* | 8.191 | 50.000 |
| 2 | grassland | ambient | ambient | *Anthemis arvensis* | 10.597 | 2.041 |
| 2 | grassland | ambient | ambient | *Cerastium glomeratum* | 17.313 | 34.694 |
| 2 | grassland | ambient | ambient | *Erodium moschatum* | 44.475 | 14.286 |
| 2 | grassland | ambient | ambient | *Geranium dissectum* | 80.090 | 10.204 |
| 2 | grassland | ambient | ambient | *Sinapis alba* | 15.553 | 38.776 |
| 2 | grassland | reduced | ambient | *Capsella bursa pastoris* | 94.288 | 10.526 |
| 2 | grassland | reduced | ambient | *Cerastium glomeratum* | 15.868 | 34.211 |
| 2 | grassland | reduced | ambient | *Lamium amplexicaule* | 46.140 | 2.632 |
| 2 | grassland | reduced | ambient | *Sinapis alba* | 14.844 | 52.632 |
| 2 | grassland | ambient | increased | *Cerastium glomeratum* | 16.543 | 57.895 |
| 2 | grassland | ambient | increased | *Geranium dissectum* | 54.799 | 10.526 |
| 2 | grassland | ambient | increased | *Sinapis alba* | 16.529 | 26.316 |
| 2 | grassland | reduced | increased | *Cerastium glomeratum* | 17.068 | 43.750 |
| 2 | grassland | reduced | increased | *Erodium moschatum* | 36.333 | 18.750 |
| 2 | grassland | reduced | increased | *Geranium dissectum* | 103.326 | 12.500 |
| 2 | grassland | reduced | increased | *Senecio vulgaris* | 8.932 | 25.000 |
